# Genomic and Transcriptomic Analyses of Malignant Pleural Mesothelioma (MPM) Samples Reveal Crucial Insights for Preclinical Testing

**DOI:** 10.3390/cancers15102813

**Published:** 2023-05-18

**Authors:** Alexander Laure, Angelica Rigutto, Michaela B. Kirschner, Lennart Opitz, Linda Grob, Isabelle Opitz, Emanuela Felley-Bosco, Stefanie Hiltbrunner, Alessandra Curioni-Fontecedro

**Affiliations:** 1 Faculty of Science and Medicine, University of Fribourg, CH-1700 Fribourg, Switzerlandstefanie.hiltbrunner@unifr.ch (S.H.); 2 Faculty of Science, University of Zurich, CH-8006 Zurich, Switzerland; 3 Department of Thoracic Surgery, University Hospital Zurich, CH-8091 Zurich, Switzerland; 4 Functional Genomics Center Zurich, Swiss Federal Institute of Technology, University of Zurich, CH-8057 Zurich, Switzerland; lennart.opitz@fgcz.ethz.ch; 5 NEXUS Personalized Health Technologies, ETH Zurich, CH-8092 Zurich, Switzerland; grob@nexus.ethz.ch; 6 Swiss Institute of Bioinformatics, CH-1015 Lausanne, Switzerland; 7 Laboratory of Molecular Oncology, Department of Thoracic Surgery, University Hospital Zurich, CH-8091 Zurich, Switzerland; 8 Department of Medical Oncology and Haematology, University Hospital Zurich, CH-8091 Zurich, Switzerland; 9 Department of Oncology, HFR Fribourg-Hôpital Cantonal, CH-1708 Fribourg, Switzerland

**Keywords:** malignant pleural mesothelioma, patient-derived cell lines, preclinical models, transcriptomic analysis, genomic analysis

## Abstract

**Simple Summary:**

Cell lines serve as tools for understanding molecular signatures of predictive biomarkers. However, the use of highly passaged commercial cell lines has to be questioned, as these do not closely resemble the originating tumor. Here, we established patient-derived cell lines from MPM tumors and performed whole exome and mRNA sequencing to understand genomic and transcriptomic differences between MPM tumors, patient-derived cell lines, and commercial cell lines. Our results show that the genome and transcriptome of tumors correlate to a higher degree with patient-derived cell lines than with commercial cell lines. These findings are of major relevance for the scientific community in regard to using cell line models to study predictive biomarkers and to interpret preclinical results which cannot be translated in clinical practice.

**Abstract:**

Cell lines are extensively used to study cancer biology. However, the use of highly passaged commercial cell lines has to be questioned, as they do not closely resemble the originating tumor. To understand the reliability of preclinical models for Malignant pleural mesothelioma (MPM) studies, we have performed whole transcriptome and whole exome analyses of fresh frozen MPM tumors and compared them to cell lines generated from these tumors, as well as commercial cell lines and a preclinical MPM mouse model. Patient-derived cell lines were generated from digested fresh tumors and whole exome sequencing was performed on DNA isolated from formalin-fixed, paraffin-embedded (FFPE) tumor samples, corresponding patient-derived cell lines, and normal tissue. RNA sequencing libraries were prepared from 10 fresh frozen tumor samples, the 10 corresponding patient-derived cell lines, and 7 commercial cell lines. Our results identified alterations in tumor suppressor genes such as *FBXW7*, *CDKN2A*, *CDKN2B*, and *MTAP*, all known to drive MPM tumorigenesis. Patient-derived cell lines correlate to a high degree with their originating tumor. Gene expressions involved in multiple pathways such as EMT, apoptosis, myogenesis, and angiogenesis are upregulated in tumor samples when compared to patient-derived cell lines; however, they are downregulated in commercial cell lines compared to patient-derived cell lines, indicating significant differences between the two model systems. Our results show that the genome and transcriptome of tumors correlate to a higher degree with patient-derived cell lines rather than commercial cell lines. These results are of major relevance for the scientific community in regard to using cell lines as an appropriate model, resembling the pathway of interest to avoid misleading results for clinical applications.

## 1. Introduction

Malignant pleural mesothelioma (MPM) is an aggressive and rare tumor with a poor prognosis arising from the pleural lining. For inoperable MPM, the current standard of care is a platinum-based chemotherapy or an immunotherapy combination, the later showing a benefit in overall survival [1,2,3]. Despite several approaches, no second line treatment has been shown to improve survival for patients with MPM; therefore, inclusion into clinical trials is recommended with an urgent need for new treatment options.

To study tumor biology and to discover targets for treatment, it is crucial to use reliable preclinical models. For cancer research, these include cell lines, 3D organoids, or systems in living organisms, in particular rodents (summarized in [4]). Syngeneic mouse models with subcutaneously injected cell lines are widely used due to the reproducible tumor take, visible tumor formation, and the fact that they have been extensively used to test new treatments for MPM (summarized in [5]).

Preclinical testing of drugs on cancer cell lines is a common practice to study cancer biology and molecular pharmacology. However, the relevance of highly passaged commercial cell lines has to be questioned, as they might not closely resemble the originating tumor [6]. In lung cancer cell lines, for example, the transcriptome varies drastically when compared to the original tumor, although the genetic and epigenetic status remain the same [7]. A comparison of cell lines to originating tumors including tumor cell enrichment to study the transcriptome and whole exons sequencing is still lacking for MPM.

Extensive studies investigating transcriptomic and proteomic changes have been performed in various cancer types, such as breast cancer, ovarian cancer, glioblastoma, and hepatocellular carcinoma, indicating significant differences between commercial cancer cell lines and patient-derived (low-passage-number) cell lines compared to the originating tumor material [8,9,10,11,12]. Transcriptomic analysis of human mesothelioma tissue focuses on differentially expressed gene signatures as prognostic markers in MPM revealing an upregulation of genes related to cell cycle processes, DNA repair, chromosome organization, epithelial–mesenchymal transition (EMT), telomere organization, and proliferation in patients with a poor prognosis [13,14,15]. However, a comparison between patient-derived cell lines and matching tumor samples for these pathways is scarce in MPM [16]. Therefore, it is unclear how closely they resemble the human disease.

The aim of this study was to compare transcriptomic and genomic profiles of patient-derived cell lines and their originating tumors with commercial cell lines in order to understand which model system should be used to answer which research question. Moreover, we analyzed the transcriptome of a syngeneic MPM mouse model and correlated this to the human disease. Our data will help to understand the mesothelioma biology and how to choose appropriate preclinical models for future studies.

## 2. Materials and Methods

### 2.1. Patient Characteristics

Tumor tissue and clinical data were collected from 10 patients undergoing surgical resection before the start of chemotherapy between 2018 and 2020 at the University Hospital Zurich. Patients included in this study had a median age of 66 years (range 64–90 years). Nine (90%) male and one (10%) female patient were included with seven (70%) epithelioid and three (30%) biphasic histological subtypes. The progression free survival (PFS) from the start of treatment was 10.6 months (range 5.62–18.63 months) and the average overall survival (OS) was 13.88 months (range 6.24–38.73 months) (Table S1). All patients gave informed, written consent and the study was conducted in accordance with the declaration of Helsinki. The study was approved by the Ethical Committee of the Canton of Zurich under reference number EK-ZH 2020-02566.

### 2.2. Human Commercially Available Mesothelioma Cell Lines

The following commercial cell lines were used in this study: MSTO-211 (ATCC CRL-2081), NCI-H28 (ATCC CRL-5820), Mero82 (ECACC 09100105), ACC-MESO1 (RCB RCB2292), ONE58 (ECACC 10092313), ZL55 (ECACC 12050301), and the MeT-5A control cell line (ATCC CRL-9444). Cells were cultured in a humidified incubator at 37 °C and 5% CO_2_ in DMEM (ZL55, Mero82 and MeT-5A) or RPMI 1640 (MSTO-211H, NCI-H28, ONE58, and ACC-MESO1) supplemented with 10% heat-inactivated FBS (all from Dominique Dutscher, Brumath, France), 2 mM L-glutamine, 50 units Penicillin, and 50 µg/mL Streptomycin (all from Gibco, Billings, MT, USA). All cell lines were negatively tested for mycoplasma using the VenorGeM Mycoplasma PCR detection kit (Sigma-Aldrich, St. Louis, MO, USA) according to the manufacturer’s instructions.

The patient-derived cell lines used in this study, MPM659 (biphasic), MPM665 (biphasic), MPM671 (epithelioid), MPM673 (epithelioid), MPM680 (epithelioid), MPM686 (epithelioid), MPM690 (epithelioid), MPM693 (biphasic), MPM700 (epithelioid), and MPM716 (epithelioid), were established in our laboratory.

### 2.3. Generation of Patient-Derived Cell Lines

Tumor tissue was cut into 3 × 3 mm pieces and either frozen in FBS containing 10% DMSO and stored in liquid nitrogen until further use or digested using the human Tumor Dissociation Kit (Miltenyi Biotec, Bergisch Gladbach, Germany) according to the manufacturer’s instructions. In short, tumor pieces were incubated with the enzyme mix in RPMI 1640 three times for 30 min at 37 °C with thorough shaking by hand in between. After the incubation, the remaining tissue was smashed through a 100 µm cell strainer and the cells were washed with RPMI 1640 supplemented with 10% FBS, 2 mM L-glutamine, 50 units Penicillin, 50 µg/mL Streptomycin, 25 mM HEPES (Sigma-Aldrich), 1× MEM Non-Essential Amino Acids Solution (Gibco) (MPM media), and 0.25 µg/mL Amphotericin B (Gibco). The cell suspension was centrifuged at 450× *g* for 5 min and plated out in 10 cm collagen-coated dishes (Cellcoat, Sheffield, UK). Amphotericin B (Life Technologies, Carlsbad, CA, USA) was supplemented until passage two. After the third passage, Human FibrOut 1 (CHI Scientific, Maynard, MA, USA) was supplemented into the MPM medium to deplete fibroblast contaminations.

### 2.4. Immunocytochemistry

Patient-derived cell lines were seeded in 8-well µ-Slides (Ibidi, Gräfelfing, Germany, 80826) to check for potential fibroblast contaminations. After 72 h of incubation, the slides were washed with TBS, fixed for 10 min in Histofix (Carl Roth, Karlsruhe, Germany), blocked for 2 h at room temperature (RT) with 10% goat serum (Abcam, Cambridge, UK), and incubated with a rabbit anti-Calretintin antibody (Acris Antibodies, San Diego, CA, USA, DRM010, 1:100) and a mouse anti-TE7-fibroblast antibody (Novus Biologicals, Centennial, CO, USA, NBP2-50082, 1:100) over night at 4 °C. The next day the slides were incubated with a goat anti-rabbit AlexaFluor594 antibody (Abcam, ab150088, 1:500) and a goat anti-mouse AlexaFluor488 antibody (Abcam, ab150117, 1:500) for 2 h at RT. The slides were mounted with VECTASHIELD Vibrance Antifade Mounting Medium containing DAPI (Vector Laboratories, Newark, CA, USA), scanned on a CLSM–Leica SP8 CLSM, and analyzed using Fiji (version 2.9.0) [17]. Cell lines positive for the fibroblast marker TE7 and negative for Calretinin were excluded from further analysis (Figure S1a). A total of 10 cell lines could be characterized as fibroblast negative and were used for further experiments, resulting in an average success rate of establishing patient-derived cell lines of about 60%.

### 2.5. Immunohistochemistry (IHC)

Four µm thick sections were cut from paraffin blocks and mounted on positively charged glass slides. The tissue was stained for Calretinin (CELL MARQUE, Rocklin, CA, USA, 5992184001, prediluted), Mesothelin (Spring Bioscience, Pleasanton, CA, USA, M3742, 1:100), NF2 (Sigma, HPA003097, 1:100), and Podoplanin (DAKO, M3619, 1:200) using an automated IHC platform (Ventana Benchmark). Stainings were performed using a pretreatment of 30 min with H_2_O_2_, a primary antibody incubation for 30 min at RT, and heat-induced epitope retrieval using HIER2 solution with a Bond Polymer Refine Detection kit (Leica Biosystems, Wetzlar, Germany). The slides were imaged using a Zeiss Axio Scan.Z1 and analyzed using QuPath (version 0.4.2), tumor regions were assigned using H&E stainings, and positive tumor cells were grouped based on DAB (3,3′-Diaminobenzidine) staining intensity into low, medium, and high expressing cells.

### 2.6. Western Blot

Protein extracts were obtained by lysing cells in TNN buffer supplemented with 1× Complete ULTRA protease inhibitor (Roche, Basel, Switzerland) and 1× PhosSTOP (ThermoFisher Scientific, Waltham, MA, USA). A total of 25 µg protein was separated on 4–10% Criterion TGX Stain-Free Protein Gels (BioRad, Hercules, CA, USA) or Mini-PROTEAN TGX Stain-Free Protein Gels (BioRad) and immediately transferred to Trans-Blot Turbo Midi PVDF Transfer Packs (BioRad) or Trans-Blot Turbo Mini Transfer Packs (BioRad). The blots were blocked with 5% nonfat dry milk in Tris-buffered saline with Tween-20 (TBST) for 1 h at RT followed by incubation with anti-human CDKN2A antibody (Abcam, ab108349, 1:1000), anti-human Mesothelin antibody (Abcam, ab93620, 1:150), anti-human Podoplanin antibody (Abcam, ab236529, 1:500), and anti-human NF2 antibody (Abcam, ab109244, 1:10,000) overnight at 4 °C. The detection antibody HRP anti-rabbit IgG (BioLegend, San Diego, CA, USA, 410406, 1:1000) was incubated for 1 h at RT. Membranes were imaged using either Clarity Western ECL Substrate (Biorad) or SuperSignal West Femto Maximum Sensitivity Substrate (ThermoFisher Scientific) on a Fusion FX (Vilber Lourmat, Eberhardzell Germany). GAPDH and α-Tubulin were used as loading controls, anti-human GAPDH antibody (ThermoFisher Scientific, MA5-15738, 1:1000), and anti-human α-Tubulin antibody (Sigma-Aldrich, T5168, 1:1000).

### 2.7. Whole Exome Sequencing

DNA was isolated from 2–3 0.6 mm punches from tumor and healthy tissue regions from formalin-fixed, paraffin-embedded (FFPE) blocks at the Institute of Pathology and Molecular Pathology at the University Hospital Zürich using the Maxwell 16 FFPE Tissue LEV DNA Purification Kit (Promega, Madison, WI, USA). DNA was further isolated from patient-derived cell lines using the PureLink Genomic DNA Mini Kit (Invitrogen, Waltham, MA, USA). Library preparation and sequencing was performed at the Genomics Facility Basel. Initial DNA quality controls were performed using the Agilent NGS FFPE QC Kit (Agilent, Santa Clara, CA, USA) and a Genomic DNA ScreenTape Analysis (Agilent). FFPE samples were treated with the NEBNext FFPE DNA Repair v2 Module (NEB). Sequencing libraries were prepared using the Human All Exon V6+UTRs kit (Agilent) according to the manufacturer’s protocol (SureSelect XT Automated Target Enrichment for Illumina Paired-End) including QC steps of DNA shearing, pre-capture libraries, post-capture libraries, and pooling using the HS NGS Fragment Kit (1–6000 bp) (Agilent). Sequencing was performed on a NovaSeq6000 Sequencing system with one lane of a S4 FlowCell v1.5 with a 200 Cycle reagent kit v1.5 (all from Illumina, San Diego, CA, USA).

The nf-core/sarek pipeline (version 3.0.1) [18] written in Nextflow (version 22.10.6.) [19] was used in conjunction with Singularities (version 3.8.3.) [20] to perform the variant calling. In brief, the pipeline performed a preprocess using bwa (version 0.7.17) [21] to conduct the alignment of the reads to the reference genome GRCh38. Duplicated reads were removed in post-processing. Somatic variants were called using the GATK4 tool Mutect2 and the healthy tissue samples were used to identify germline mutations (version 4.2.6.1) [22]. Copy number variants were identified using CNVkit and Control-FREEC (version 0.9.9) and only union results were used [23,24].

### 2.8. Murine RN5 Model

Eight-week-old female C57BL/6 mice were purchased from Janvier Labs and housed under pathogen-free conditions at the Laboratory Animal Service Center (LASC) of the University of Zürich. All animal experiments were performed in accordance with the Swiss federal and cantonal regulations on animal protection and were approved by The Cantonal Veterinary Office Zurich (ZH147/2018).

Murine mesothelioma RN5 cells were cultured in a humidified incubator at 37 °C and 5% CO_2_ in DMEM supplemented with 10% heat-inactivated FBS, 2 mM L-glutamine, 50 units Penicillin, and 50 µg/mL Streptomycin [25]. Cells were negatively tested for Mycoplasma using the VenorGeM Mycoplasma PCR detection kit. For tumor analysis, 2 × 10^6^ RN5 cells were injected s.c. in the flank of C57BL/6 mice. Mice were euthanized after tumors reached a size of 800 mm^3^.

### 2.9. Tumor Cell Isolation from Human and Mouse Tumors for RNA Sequencing

Ten human MPM tumors and six murine RN5 tumors were dissociated using the human Tumor Dissociation Kit (Miltenyi Biotec) for human samples and 600 U/mL DNAse 1 (Roche) and 100 mg/mL Collagenase 1 for murine samples. Tumor cell suspensions were depleted from CD31^+^ endothelial cells, CD45^+^ immune cells, and Ter119^+^ erythrocytes using the human/mouse Tumor Cell Isolation Kit (Miltenyi Biotec) according to the manufacturer’s instructions. In brief, single cell suspensions were incubated with 20 µL Non-Tumor Cell Depletion Cocktail A and 20 µL Non-Tumor Cell Depletion Cocktail B for 15 min at 4 °C. After incubation, the total reaction volume was adjusted to 1 mL using PBS supplemented with 0.5% bovine serum albumin (Roth) and the suspension was loaded on pre-activated LS Columns (Miltenyi Biotec) on a magnetic rack. The tumor cells in the flow through were collected and used for RNA isolation. The isolation purity was analyzed by flow cytometry using anti-human CD45-FITC antibody (Miltenyi Biotec, 130-110-769, 1:50), anti-human CD31-PE antibody (Miltenyi Biotec, 130-110-807, 1:50) (Figure S1b), anti-mouse CD45-FITC antibody (Miltenyi Biotec 130-110-796, 1:50), and anti-mouse CD31-PE antibody (Miltenyi Biotec, 130-111-540, 1:50) (Figure S1c). Dead cells were excluded using the LIVE/DEAD Fixable Violet Dead Cell Stain Kit (Invitrogen). All samples were acquired on a LSR II Fortessa 4L and data were analyzed using FlowJo (version 10.0.8).

### 2.10. RNA Sequencing

Total RNA was isolated from cell line pellets containing 10^6^ cells and tumor samples after magnetic tumor cell isolation using the RNeasy Mini Kit (Qiagen, Venlo, Netherlands). The quality of the isolated RNA was determined using a Fragment Analyzer (Agilent). Samples with a 260 nm/280 nm ratio between 1.8 and 2.1 and a 28S/18S ratio between 1.5 and 2 were further processed. The TruSeq Stranded mRNA protocol (Illumina) was used in the succeeding steps for murine samples. Briefly, total RNA samples (100–1000 ng) were poly-A-enriched and reverse-transcribed into double-stranded cDNA. The cDNA samples were fragmented, end-repaired, and adenylated before ligation of TruSeq adapters containing unique dual indices (UDI) for multiplexing. Fragments containing TruSeq adapters on both ends were selectively enriched with PCR. The quality and quantity of the enriched libraries were validated using the Fragment Analyzer (Agilent). The product is a smear with an average fragment size of approximately 260 bp. The libraries were normalized to 10 nM in Tris-Cl 10 mM, pH8.5 with 0.1% Tween 20.

The Novaseq 6000 (Illumina) was used for cluster generation and sequencing. Sequencings were paired-end at 2 × 150 bp or single-end 100 bp. The human libraries were prepared following the Smart-seq2 protocol [26]. Following reverse transcription, the quality of the cDNAs was evaluated using an Agilent 2100 Bioanalyzer. An amount of 0.5 ng of cDNA from each sample was tagmented and amplified using an Illumina Nextera XT kit. The resulting libraries were pooled, double-sided size selected (0.5× followed by 0.8× ratio using Beckman Ampure XP beads), and quantified using an Agilent 4200 TapeStation System. The pool of libraries was sequenced in an Illumina NovaSeq6000 sequencer (single-end 100 bp) with a depth of around 20 Mio reads per sample.

### 2.11. RNA Sequencing Analysis and Statistics

The raw reads were first cleaned by removing adapter sequences, trimming low quality ends, and filtering reads with low quality (phred quality < 20) using Fastp (version 0.20) [27]. The read alignment was performed using STAR (version 2.7.4a) [28]. As a reference we used the Ensembl genome build GRCh38.p13 with the gene annotations downloaded on 5 November 2019 from Gencode (release 32) for human samples and the Ensembl genome build GRCm38.p6 with the gene annotations downloaded on 5 November 2019 from Gencode (release M23) for murine samples. The STAR alignment options were “—outFilterType BySJout —outFilterMatchNmin 30—outFilterMismatchNmax 10—outFilterMismatchNoverLmax 0.05—alignSJDBoverhangMin 1—alignSJoverhangMin 8—alignIntronMax 1,000,000—alignMatesGapMax 1,000,000—outFilterMultimapNmax 50”.

Gene expression values were computed using the function featureCounts from the R package Rsubread (version 2.8.2) [29]. The options for featureCounts were “—min mapping quality 10—min feature overlap 10bp—count multi-mapping reads—count only primary alignments—count reads also if they overlap multiple genes”.

Differentially expressed (DE) genes were identified using the R package edgeR (version 3.36.0) [30] from Bioconductor, using a generalized linear model (glm) regression, a quasi-likelihood (QL) differential expression test, and the trimmed means of M-values (TMM) normalization. Clustering of results was performed using base R and the inertia method to identify distinct clusters [31].

### 2.12. Deconvolution of Bulk RNA Sequencing Data Using Granulator

To identify contaminations of the bulk sequencing data, deconvolution was performed using the granulator package for R (version 1.2.0) [32]. The package is used to run multiple deconvolution algorithms using reference data from single cell experiments to estimate the proportions of specific cell types in a bulk sequencing experiment. Reference gene expression data were used from Monaco et al. [33] for immune cell subtypes and from Gordon et.al [14] for healthy lung and pleura. For the final deconvolution, the non-negative least squares (nnls) algorithm was used (Figure S1d). Cells were grouped into immune cells, normal lung cells, normal pleura cells, MPM cells based on previously published signatures, and other tumor cells, showing less of the MPM phenotype but clear tumor signatures. One cell line (MPM692) was excluded from further analysis due to a high contamination of healthy tissue. All analysis steps were performed in RStudio (version v1.4.1103-4) using R (version 4.0.4).

### 2.13. Gene Set Enrichment Analysis (GSEA)

Genes were ordered based on their difference expression and enrichment scores (ES) were calculated for every predefined gene set and normalized to all dataset permutations to obtain the normalized enrichment score (NES). Here, we used the Hallmark gene sets [34] to identify differentially regulated biological states and processes. EMT regulation was analyzed using gene sets retrieved from the EMTome project [35]. Eighty-four previously published EMT signatures were compared and normalized to fresh MPM tumor samples. Venn diagrams for the comparison of genes between human and mice were made using the VennDiagram (version 1.7.3) R package.

## 3. Results

### 3.1. Characterization of MPM Tumors

**Figure 1:**
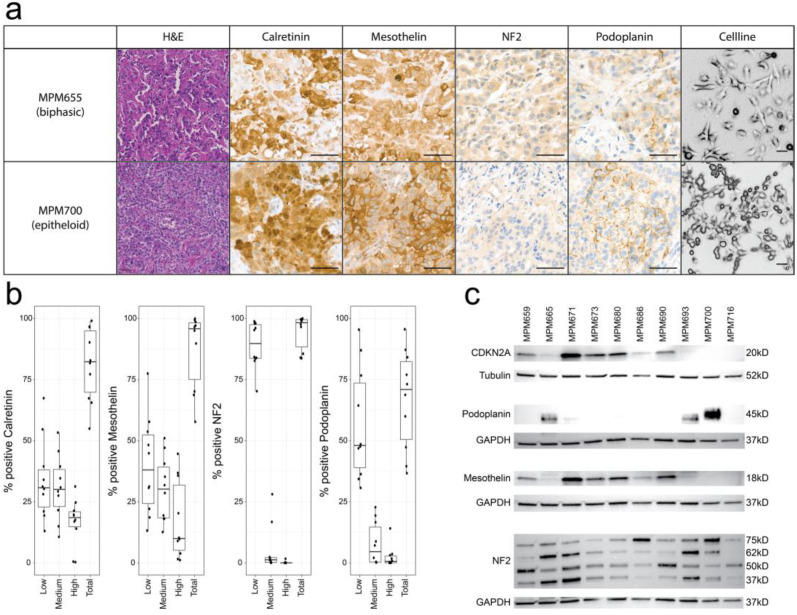
Characterization of MPM tumors and patient-derived cell lines: (**a**) Representative H&E stainings and IHC stainings for Calretinin, Mesothelin, NF2, and Podoplanin of MPM665 and MPM700. Cell line images of the corresponding cell line were taken to analyze their morphology. H&E and cell line 100 µm scale bar; Calretinin, Mesothelin, NF2, and Podoplanin 50 µm scale bar. (**b**) Quantification of IHC stainings; tumor cells were grouped according to low, medium, and high expression of the specific marker on tumor cells. Calretinin is expressed in 79.2% ± 15.7% tumor cells, Mesothelin in 87.2% ± 14.3%, Podoplanin, in 60.8% ± 23.6%, and NF2 in 93.1% ± 8.08%. (**c**) Western blots of CDKN2A, Podoplanin, Mesothelin, and NF2 for patient-derived cell lines including respective loading controls (Original western blots are presented in Table S7).

We characterized all human MPM tumors, which were used to establish patient-derived cell lines: MPM671, MPM673, MPM680, MPM68, MPM690, MPM700, and MPM716 were identified as epithelioid and MPM665, MPM659, and MPM693 as biphasic based on hematoxylin and eosin (H&E) staining (Figure 1a). The expression of MPM markers was analyzed using IHC and positive tumor cells were quantified: all tumors were positive for Calretinin, Mesothelin, and Podoplanin, with an average of positive tumor cells/sample of 79%, 87%, and 61%, respectively (Figure 1b). It has been previously described that about 30% of MPM tumors harbor an alteration in the *NF2* gene [36] which causes loss of expression or a short variant of the encoded gene Merlin. Merlin-expressing tumor cells were detected in all tumor samples (93.1% ± 8.08%). As the antibody targets the short variant of NF2, we have performed WES to evaluate the mutational status of this gene.

To characterize the established patient-derived cell lines, Western blots for the MPM markers Mesothelin and Podoplanin were performed (Figure 1c). Mesothelin expression was found in 60% of the cell lines, Podoplanin in 30%. Four cell lines lacked the expression of the tumor suppressor CDKN2A and NF2 variants could be found in every cell line, indicating no deep deletions of this gene in the patient-derived cell lines. Isoforms and dimer variants of Merlin, the protein encoded by *NF2*, were found in all patient-derived cell lines.

### 3.2. Genomic Profiling Identifies MPM Specific Overlapping Genetic Mutations in Patient-Derived Cell Lines and Originating Tumors

**Figure 2:**
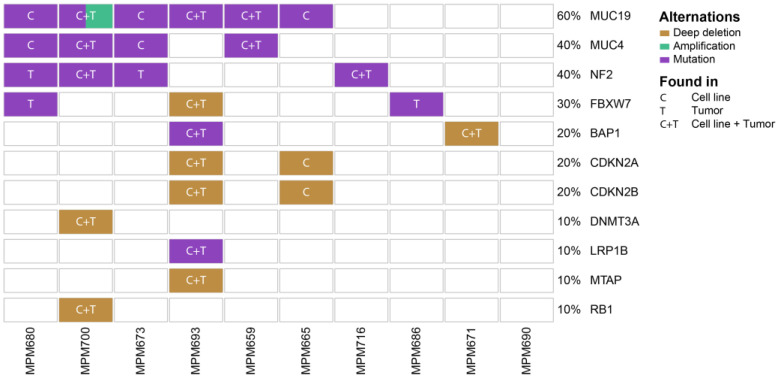
Oncoplot displays the somatic mutations distribution of the top highly mutated genes in patient-derived MPM cell lines and the corresponding tumors. Each column represents a patient. Each row represents a gene involved in MPM pathogenesis with a different variant classification. Different types of mutations are color coded. C = only cell line, T = only tumor, and C+T = cell line and tumor.

The genomic landscape of MPM is characterized by mutations in tumor suppressor genes and the loss of whole chromosome regions. We performed whole exome sequencing of 10 patient-derived cell lines and their corresponding tumor tissue and identified 1339 somatic mutations present in at least one sample with 130 genes present in at least two samples (Table S2). A total of 1013 single nucleotide variants (SNVs) were associated with missense variants, 69 with frameshift variants, 69 with stop gains, and 56 with in-frame deletions. SNVs were further categorized based on their impact on the translated protein. A total of 16% of all SNVs have a high impact, 74% medium, and 10% low or no impact on the functional protein. Genes were further filtered based on the COSMIC cancer gene census list of genes [37]. These genes are frequently mutated in cancer and are associated with tumor development and progression. Six of those genes were found to be mutated in at least one sample. *MUC19* contains missense mutations in six patient-derived cell lines, which were also found in three corresponding tumors. An amplification of *MUC19* could be found in MPM700. *MUC4* missense mutations were identified in four patient-derived cell lines and two corresponding tumors. *NF2* mutations leading to short variants and impaired protein function were found in two patient-derived cell lines and their corresponding tumors and two additional tumors. *FBXW7* mutations were identified in three tumors and one corresponding cell line. *BAP1* and *LRP1B* mutations were found in the MPM693 tumor and corresponding patient-derived cell line (Figure 2).

Deep deletions of chromosome regions containing *FBXW7*, *CDKN2A*, *CDKN2B*, and *MTAP* could be identified in the MPM693 patient-derived cell line and the corresponding tumor. *DNMT3A* and *RB1* are deleted in the MPM700 cell line and tumor. *CDKN2A* and *CDKN2B* deletions were found in the MPM665 patient-derived cell line but not in the corresponding tumor. A *BAP1* deletion was found in the MPM671 cell line and tumor. The patient-derived cell lines MPM659, MPM671, MPM693, MPM700, and MPM716 harbor the same mutational changes in tumor genes when compared to their corresponding tumor.

To understand if genomic alterations can be predicted on a transcriptomic level, we analyzed the expression of *BAP1*, *CDKN2A*, *CDKN2B*, *DNMT3A*, *FBXW7*, *LRP1B*, *MTAP*, *MUC19*, *MUC4*, *RB1*, and *NF2* in unmutated samples compared to samples with missense mutations and deletions. Although higher expression can be observed in unmutated samples for all genes, the changes are only significant for *BAP1*, *CDKN2B*, *RB1*, and *NF2* (Figure S2).

In summary, this analysis identified somatic mutations and deletions in tumor suppressor genes known to drive MPM tumorigenesis. The patient-derived cell lines correlate to a high degree with their originating tumor and can be used as in vitro models to study mesothelioma with different genomic backgrounds. No germline driver mutations according to the COSMIC database could be identified in the analyzed samples.

### 3.3. Gene Expression Profiles of MPM Tumors, Patient-Derived Cell Lines, and Commercial Cell Lines form Distinct Clusters

**Figure 3:**
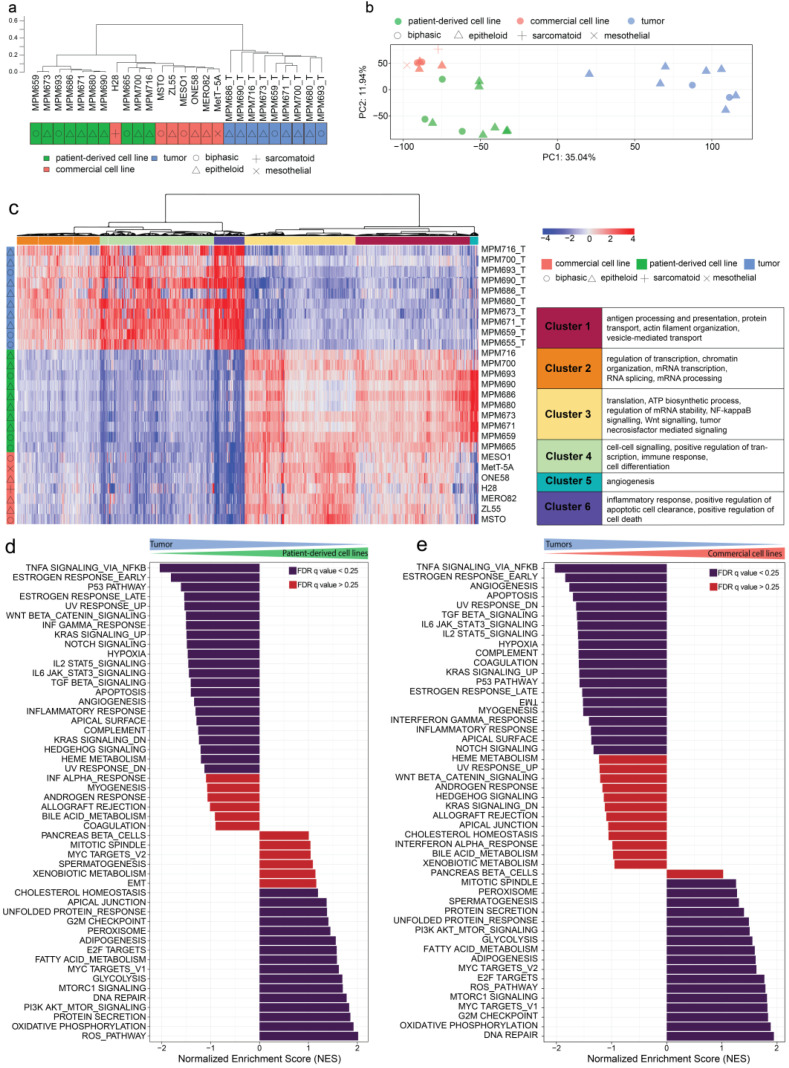
Transcriptome analysis of patient-derived MPM cell lines, commercial MPM cell lines, and MPM tumor material. (**a**) Hierarchical clustering of all samples using the top 100 differentially expressed genes shows 3 distinct clusters representing samples from different origins and the non-cancerous Met-5A cell line clustering the furthest away from all other samples. (**b**) Three distinct clusters could be further validated using PCA analysis across all genes and all samples (Q = 0.6841). PC1 represents 35% of variance and PC2 12%. (**c**) Differential gene expression analysis with a significance threshold of 0.01 and a log2 ratio threshold of 0.5 between cell lines and tumors shows a downregulation of processes involved in antigen processing and presentation, protein transport, actin filament organization and vesicle-mediated transport, translation, the ATP biosynthetic process, regulation of mRNA stability, NIK/NF-κB signaling, Wnt signaling, the tumor necrosis factor-mediated signaling pathway, and angiogenesis in tumors compared to cell lines. GO terms related to regulation of transcription, chromatin organization, mRNA transcription, RNA splicing, mRNA processing, cell–cell signaling, positive regulation of transcription, immune response, cell differentiation, inflammatory response, positive regulation of apoptotic cell clearance, and positive regulation of cell death were downregulated in cell lines compared to tumors. (**d**) Gene set enrichment analysis (GSEA) for Hallmark gene sets for tumors vs. patient-derived cell lines and tumors vs. commercial derived cell lines (**e**). Blue bars indicate significant enrichment between groups with FDR *q* values < 0.25. Red bars indicate non-significant FDR *q* values > 0.25. A positive Normalized Enrichment Score (NES) indicates enrichment in patient-derived cell lines or commercial cell lines, respectively; a negative NES indicates enrichment in.

To understand differences between the transcriptomic profile of patient-derived cell lines, their originating tumor tissue, and commercial cell lines we performed whole mRNA sequencing on samples from these different origins. We analyzed ten patient-derived cell lines with matching tumors, seven commercial cell lines, and the MeT-5A control cell line. The transcriptomes were analyzed using hierarchical clustering based on the top 100 differentially expressed genes across all human samples (Tables S3–S5, Figure S3). On hierarchical clustering, the MeT-5A cell line clusters furthest away from all other samples; patient-derived and commercial cell lines clustered together while tumors clustered separately from all cell lines (Figure 3a). A principal component analysis (PCA) identified three distinct clusters (Q = 0.6841) representing the three sorts of samples (commercial, patient-derived cell lines, and tumors) (Figure 3b). Here, commercial cell lines showed transcriptional profiles which differ from MPM tumors with specific features. To understand the differences between commercially available cell lines, patient-derived cell lines, and tumors, we performed further differential pathway analysis.

### 3.4. Differential Gene Expression Analysis Reveals Upregulation of Metabolic and Cell-Cycle-Related Processes in Cell Lines Compared to an Upregulation of Genes Involved in Transcription, EMT, and Immune System Response in Tumors

We analyzed differently expressed genes between commercial, patient-derived cell lines, and originating tumors with a significance threshold of 0.01 and a log2 ratio threshold of 0.5. The analysis revealed 4235 expressed genes, with 6 distinct clusters based on the 2000 top differentially expressed genes (Figure 3c). Cluster 1 includes Gene Ontology (GO) terms related to antigen processing and presentation (GO:0019886), protein transport (GO:0019886), actin filament organization (GO:0019886), and vesicle-mediated transport (GO:0019886). Cluster 2 includes GO terms related to regulation of transcription (GO:0006355), chromatin organization (GO:0006325), mRNA transcription (GO:0042789), RNA splicing (GO:0008380), and mRNA processing (GO:0006397). Cluster 3 includes the GO terms translation (GO:0006412), ATP biosynthetic process (GO:0006754), regulation of mRNA stability (GO:0043488), NIK/NF-κB signaling (GO:0038061), Wnt signaling (GO:0060071), and tumor necrosis factor-mediated signaling pathway (GO:0033209). Cluster 4 includes the GO terms cell–cell signaling (GO:0007267), positive regulation of transcription (GO:0045893), immune response (GO:0006955), and cell differentiation (GO:0030154). Cluster 5 includes the GO term angiogenesis (GO:0001525). Cluster 6 includes the GO terms inflammatory response (GO:0006954), positive regulation of apoptotic cell clearance (GO:2000427), and positive regulation of cell death (GO:0010942). Clusters 1, 3, and 5 were significantly downregulated in tumor tissue compared to all cell lines. Clusters 2, 4, and 6 were downregulated in cell lines compared to tumors.

These results indicate an upregulation of metabolic and cell-cycle-related processes in cell lines compared to an upregulation of genes involved in transcription, epithelial-mesenchymal transition (EMT), and immune system response in tumor tissue.

### 3.5. Transcriptomic Regulation of EMT, Apoptosis, UV-Response Inhibition, Myogenesis, and Angiogenesis Are More Comparable to Tumors in Patient-Derived Cell Lines Than Commercial Cell Lines Based on Gene Set Enrichment Analysis (GSEA)

GSEA between tumors and corresponding patient-derived cell lines with a false discovery rate (FDR) cutoff at 25% revealed a significant upregulation of gene sets including the reactive oxygen species pathway, oxidative phosphorylation, PI3K/AKT/mTOR signaling, and MYC targets in patient-derived cell lines, while tumors showed an upregulation of TNFα signaling via NF-κB, KRAS signaling, Notch signaling, TGFβ signaling, hypoxia, IL-6 JAK STAT3 signaling, IL-2 STAT5 signaling, inflammatory response, and Hedgehog signaling (Figure 3d,e).

The comparison between patient-derived and commercial cell lines (FDR < 25%) revealed an upregulation of gene sets including EMT, UV response inhibition, IL-6 JAK STAT3 signaling, IL-2 STAT5 signaling, inflammatory response, and hypoxia in patient-derived cell lines (Figure S4). In contrast, an upregulation of MYC targets, oxidative phosphorylation, and UV response occurs in commercial cell lines. These results reveal that gene expression involved in multiple pathways such as EMT, apoptosis, UV-response inhibition, myogenesis, and angiogenesis are upregulated in tumor samples when compared to patient-derived cell lines; however, they are downregulated in commercial cell lines compared to patient-derived cell lines, indicating significant differences between the two model systems with closer similarities of patient-derived cell lines to tumors for these gene sets.

### 3.6. Genes of the EMT Pathway Are Upregulated in Patient-Derived Cell Lines Compared to Commercial Cell Lines Displaying Similarities to Tumors

Due to the significant differences of EMT signaling in commercial and patient-derived cell lines and no significant differences between patient-derived cell lines and tumors, we decided to investigate genes involved in EMT regulation. We specifically investigated if specific genes involved in this pathway were regulated differentially. The reversible transition from an epithelial to a mesenchymal phenotype is accompanied by the downregulation of epithelial markers such as E-cadherin, Collagen type 6, and Cytokeratin 5/6 and an upregulation of mesenchymal markers such as α-SMA, N-cadherin, Cadherin 11, and Vimentin [38]. The transition to a mesenchymal phenotype results in increased motility, a stem-cell-like phenotype, and an increased chemotherapeutic resistance [39].

**Figure 4:**
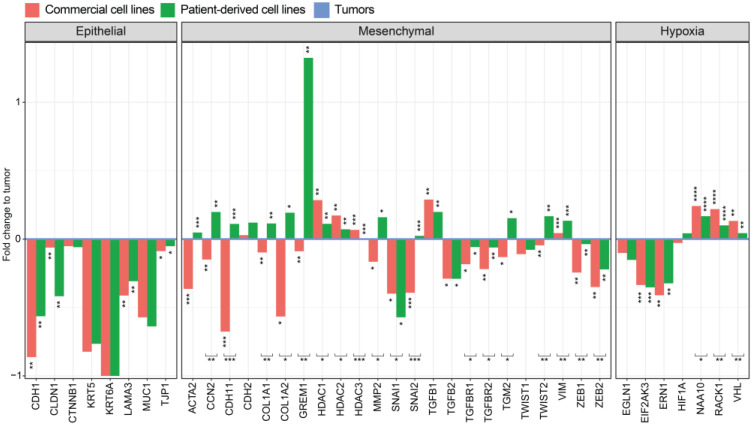
Epithelial–mesenchymal transition analysis in primary MPM tumors compared to patient-derived and commercial cell lines. Analysis of EMT genes normalized to primary tumors. Significance was calculated for patient-derived cell lines to commercial cell lines (brackets) and to primary tumors using a *t*-test and *p*-values adjusted using Bonferroni corrections. **** *p* < 0.0001, *** *p* < 0.001, ** *p* < 0.01 and * *p* < 0.05.

We compared our data with 84 previously published EMT signatures from the EMTome project [35]. The EMTome database project collects EMT core signatures from scattered literature and identifies their interactions in cancers. The analysis showed an increased mesenchymal phenotype of patient-derived cell lines compared to commercial cell lines and tumors (Figure S5). Individual genes related to an epithelial or mesenchymal phenotype were analyzed. The well-characterized epithelial marker *CDH1* is significantly downregulated in all cell lines compared to tumors, but this difference is significantly higher in commercial cell lines compared to patient-derived ones. This is also true for other known markers of an epithelial phenotype as *KRT5*, *KRT6A*, *LAMA3*, and *TJP1* [40,41] (Figure 4).

The mesenchymal markers *ACTA2*, *CDH2*, *CDH11*, and *VIM* are involved in proliferation, generation of stem-cell-like cells, and tumor progression [39,42]. These were all upregulated in patient-derived cell lines compared to tumors while downregulated in commercial cell lines.

As the main inducer of epithelial EMT, *TGFB1* is significantly upregulated in all cell lines compared to tumors. However, other members of the TGFβ signaling pathway such as *TGFB2*, *TGFBR1*, and *TGFBR2* are downregulated in all cell lines; this downregulation is significantly higher for commercial cell lines.

EMT signaling through the transcription factor *TWIST1* is decreased in all cell lines compared to a significant increase in *TWIST2* signaling only in patient-derived cell lines. This could explain the upregulation of *MMP2* in patient-derived cell lines and the overall increased mesenchymal phenotype. The mesenchymal signaling through the transcriptional repressor *SNAI1* is significantly decreased in all cell lines compared to a significant increase in *SNAI2* (Slug) signaling in patient-derived cell lines only. The transcription factors *ZEB1* and *ZEB2* are significantly downregulated in all cell lines compared to tumor tissue.

*GREM1* (Gremlin 1) is highly upregulated in patient-derived cell lines. It is known to induce an invasive phenotype in MPM through *SNAI2* [43]. *TGM2* (Transglutaminase 2) is upregulated in patient-derived cell lines and downregulated in commercial cell lines. TGM2 plays a role in tumor formation and generates a stem-cell-like phenotype in mesothelioma [44].

The subunits *COL1A1* and *COL1A2* of the mesenchymal marker collagen type IV are upregulated in patient-derived cell lines, compared to a downregulation in commercial cell lines when compared to tumors.

Histone deacetylases class 1 (*HDAC1*, *HDAC2*, *HDAC3*, and *HDAC8*) are expressed ubiquitously in almost all types of cancer except renal cancer [45]. Our data show an increased expression of HDACs class1 in commercial cell lines compared to tumor tissue and a non-significant difference between patient-derived cell lines and tumors.

These results indicate an upregulation of several EMT-inducing genes in patient-derived cell lines compared to commercial cell lines and a closer similarity of EMT regulation between tumors and the corresponding patient-derived cell lines. The results suggest that low-passage-number patient-derived cell lines should be a preferred tool to study EMT in mesothelioma.

### 3.7. Genes Involved in Oxidative Folding of Proteins Are Downregulated and Negative Regulators of Hypoxia Are Upregulated in Cell Lines Suggesting a Highly Hypoxic State in MPM Tumors

In the tumor microenvironment of most solid tumors, a state of low nutrients, high interstitial fluid pressure, and hypoxia is predominant. The lack of sufficient oxygen supply to dense tumor regions occurs due to a slower rate of angiogenesis and vascularization compared to tumor cell proliferation in cancer [46]. Hypoxia can lead to a change in cellular metabolism and affect multiple intracellular signaling pathways. The main transcriptional regulator of hypoxia is *HIF1A*, which is regulated by hydroxylation through *EGLN1* (*PHD2*). This process leads to polyubiquitination of *HIF1A* by *VHL* and results in proteasomal degradation. Further negative regulation of *HIF1A* includes *NAA10* (*ARD*) and *RACK1*.

An upregulation of the negative hypoxia regulators *NAA10*, *RACK1*, and *VHL* was observed in the transcriptome of all cell lines compared to tumor tissue, with a higher similarity between patient-derived cell lines and tumors (Figure 4), suggesting a hypoxic state in tumor tissue compared to a normoxic state in cell lines. This could be explained further by an upregulation of proliferation in cell lines, which is typically reduced in a hypoxic environment.

Furthermore, hypoxia can downregulate mTOR signaling to reduce oxygen consumption by decreasing protein synthesis [47]. Due to this downregulation, the process of oxidative folding of proteins is impaired. To overcome cell death, *EIF2AK3* (*PERK*) and *ERN1* (*IRE1*) [48] are upregulated to increase folding capacity in a hypoxic state. This upregulation of genes involved in protein folding can be seen in all cell lines when compared to tumor tissue.

For hypoxia-related genes, we could identify closer similarities of patient-derived cell lines to tumors than commercial cell lines with significance only for genes involved in negative regulation.

### 3.8. Gene Sets of the P53 Pathway, EMT, and TGFβ Signaling Are Upregulated in RN5 Tumors Compared to the RN5 Cell Line

**Figure 5:**
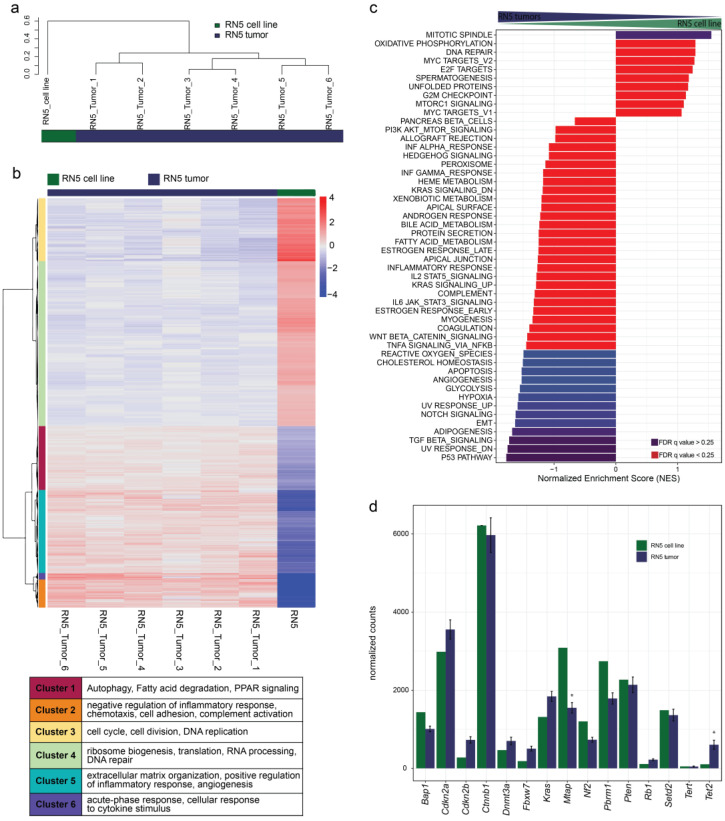
Transcriptome analysis of murine RN5 cell line and syngeneic RN5 tumor material: (**a**) Hierarchical clustering of all samples using the top 100 differentially expressed genes shows 2 distinct clusters representing samples from different origins. (**b**) Differential gene expression analysis with a significance threshold of 0.01 and a log2 ratio threshold of 0.5 between the cell line and tumors shows a upregulation of processes involved in autophagy, fatty acid metabolism, PPAR signaling, negative regulation of inflammatory response, chemotaxis, cell adhesion, complement activation, extracellular matrix organization, positive regulation of inflammatory response, angiogenesis, acute-phase response, and cellular response to cytokine stimulus in tumor tissue compared to the cell line. GO terms related to cell cycle, cell division, DNA replication, ribosome biogenesis, translation, RNA processing, and DNA repair were upregulated in cell lines compared to tumors. (**c**) Gene set enrichment analysis (GSEA) for Hallmark gene sets for RN5 tumors vs. RN5 cell line. Blue bars indicate significant enrichment between groups with FDR *q* values < 0.25. Red bars indicate non-significant FDR *q* values > 0.25. A positive Normalized Enrichment Score (NES) indicates enrichment in the cell line; a negative NES indicates enrichment in tumors. (**d**) Gene expression comparison of commonly mutated genes in the RN5 model. mRNA is shown as normalized counts of the RNA sequencing. Significance was calculated for RN5 tumors to the cell lines using a *t*-test and *p*-values adjusted using Bonferroni corrections. * *p* < 0.05.

Lastly, we were interested in the transcriptomic profile of the syngeneic mouse mesothelioma model RN5, by analyzing the RN5 cell line and the matching tumor to understand the reliability of this mouse model for preclinical testing [25]. We analyzed the transcriptomic profile from RN5 cells and compared them to tumors. To identify gene expression patterns, hierarchical clustering based on the top 100 differentially expressed genes was performed across all murine samples. We could identity two distinct clusters representing the origin of the cells (Figure 5a and Figure S6, Table S6). Analysis of differentially expressed genes between the RN5 cell line and RN5 tumors with a significance threshold of 0.01 and a log2 ratio threshold of 0.5 revealed 6046 differentially expressed genes in 6 distinct clusters (Figure 5b). Cluster 1 includes GO terms related to autophagy (GO:0006914), fatty acid metabolism (GO:0009062), and PPAR signaling (GO:0035357). Cluster 2 includes the GO terms negative regulation of inflammatory response (GO:0050728), chemotaxis (GO:0006935), cell adhesion (GO:0007155), and complement activation (GO:0006956). Cluster 3 includes GO terms related to cell cycle (GO:0007049), cell division (GO:0007049), and DNA replication (GO:0006260). Cluster 4 includes GO terms related to ribosome biogenesis (GO:0042254), translation (GO:0042254), RNA processing (GO:0006396), and DNA repair (GO:0006281). Cluster 5 includes the GO terms extracellular matrix organization (GO:0030198), positive regulation of inflammatory response (GO:0030198), and angiogenesis (GO:0030198). Cluster 6 includes the GO terms acute-phase response (GO:0006953) and cellular response to cytokine stimulus (GO:0006953). Clusters 1, 2, 5, and 6 were significantly upregulated in tumor tissue compared to the cell line. Clusters 3 and 4 were upregulated in cell lines compared to tumors.

Gene set enrichment analysis between the RN5 cell line and corresponding tumors with a FDR cutoff at 25% revealed a significant upregulation of the mitotic spindle Hallmark gene set in the cell line, while in the tumors the P53 pathway, UV response, TGFβ signaling, adipogenesis, EMT, notch signaling, hypoxia, glycolysis, angiogenesis, cholesterol homeostasis, and reactive oxygen species were upregulated (Figure 5c).

### 3.9. Genomic Alterations of the RN5 Model

To understand potential changes of genomic alterations between the RN5 cell line and RN5 tumors, we analyzed the expression of genes known to be mutated in human MPM patients. Based on our transcriptomic data, significant differences between the cell line and tumors in gene expression could only be identified for *Mtap* and *Tet2*. Low overall expression of *Bap1*, *Cdkn2b*, *Dnmt3a*, *Fbxw7*, *Nf2*, *Rb1*, *Tert*, and *Tet2* could indicate potential genomic alterations in these genes with *Bap1* and *Nf2* deficiencies previously described by Rehrauer et al. (Figure 5d) [42]. These results indicate a low mutation rate in the syngeneic mouse model and genetic alterations present in the cell line do not change over the three months of in vivo tumor growth.

### 3.10. Gene Expression Patterns Differ from Human to Murine MPM Samples

**Figure 6:**
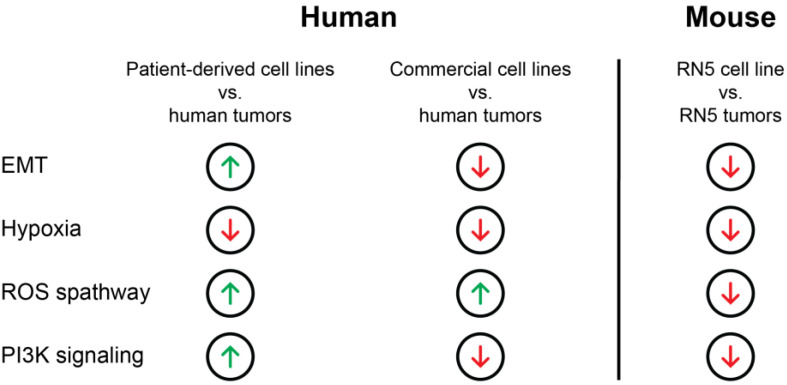
Schematic overview of the most prominent pathways regulated in humans and mice. Arrows up indicate an upregulation of the pathway in the respective cell line compared to tumors, whereas an arrow down indicates a downregulation in the cell line compared to tumors.

We further analyzed differentially expressed genes from patient-derived cell lines to tumors in humans and mice and could identify 192 genes differentially expressed in both species. A total of 46 of these genes were upregulated in both species, 109 downregulated, and 37 were differentially regulated across humans and mice (Figure S7). A total of 1598 upregulated and 1052 downregulated genes could only be found in murine tumors compared to the cell line and 260 upregulated vs. 301 downregulated genes in tumors compared to patient-derived cell lines could be found in humans. The results indicate a similar upregulation in murine samples compared to human MPM samples of metabolic and cell-cycle-related processes in the cell line compared to an upregulation of genes involved in inflammatory regulation, cell adhesion, and extracellular matrix organization in tumor tissue. The upregulation of TGFβ signaling, P53, and hypoxia in murine tumors follows the same pattern as in human MPM tumors, making this model a tool to study these pathways. However, for other pathways such as reactive oxygen species, PI3K signaling, and EMT, which are upregulated in RN5 tumors but downregulated in human MPM tumors, other preclinical models are needed (Figure 6).

## 4. Discussion

Preclinical models have largely contributed to understandings of tumor biology. Thus, the establishment and understanding of novel and relevant model systems are crucial to develop new therapeutic strategies. In our study, we aimed to comprehensively analyze transcriptomes of MPM tumors and cell lines without contamination. In order to improve the purity of the samples, we excluded fibroblasts from the patient-derived cell lines and isolated cancer cells from tumors to reduce contamination with other cell populations. Such an approach has previously been shown to improve the quality of downstream analysis [49,50]. Previous studies have compared patient-derived and commercial cell lines but lacked information for their parental tumor samples. Refs [16,51], which is crucial for translating preclinical findings into clinical applications. Kanellakis et al. evaluated the genomic and transcriptomic profiles of MPM tumors in comparison with commercial MPM cell lines only [52]. Our results reveal that patient-derived cell lines more closely resemble the originating tumors based on genomic and transcriptome alterations.

In our analysis, the patient-derived cell lines and originating tumors express MPM markers such as Calretinin, Mesothelin, and Podoplanin. However, decreased protein expression of Mesothelin and Podoplanin was found in the cell lines. It has been previously shown that MPM markers such as Calretinin are downregulated in vitro due to cell-cycle-dependent transcriptional regulation and post-transcriptional events in commercial cell lines [53,54]. CDKN2A protein expression was found in 40% of all patient-derived cell lines, indicating copy number alterations in this gene. In line with our findings, previous publications reported genomic *CDKN2A* alterations in 42% of all mesothelioma patients [36]. Isoforms and dimer variants of Merlin, the protein encoded by *NF2*, were found in all patient-derived cell lines. Two isoforms and different dimerization products of Merlin were previously identified [55]. Previous publications described that about 30% of MPM tumors harbor an alteration in the *NF2* gene [36], which causes loss of expression or a short variant of the encoded gene [56,57]. However, as the antibody used for conventional IHC also targets the short variant of *NF2*, we applied here the WES data to evaluate the mutational status of the samples.

On the molecular level, MPM tumors are characterized by genomic alterations in *BAP1* (45.1%), *CDKN2A* (42.2%), *CDKN2B* (36.0%), *NF2* (31.3%), *MTAP* (27.3%), *TP53* (17.3%), *SETD2* (10.2%), and *PBRM1* (9.1%) [36]. Here, we detected alterations in the tumor suppressor genes *BAP1* (*20%*), *CDKN2A* (*20%*), *CDKN2B* (*20%*), *MTAP* (*10%*), and *RB1* (*10%*) in patient-derived cell lines and their corresponding tumors. Alterations of *NF2* (*40%*) and *FBXW7* (*30%*) were also identified in tumors only, indicating a heterogeneity of tumor cells and clonal selection process in some patient-derived cell lines during the expansion process in vitro. A study by Ben-David et al. showed that clonal selection, rapid genetic diversification, and even gains of whole chromosome arms can occur in well-characterized commercial cell lines [58]. Moreover, highly passaged and widely used ovarian cancer cell lines have higher genomic alteration rates in vitro compared to low-passage-number cell lines [10]. Patient-derived cell lines were established from the same tumor tissue used for sequencing, to avoid samples with molecular intra-tumor heterogeneity as published before for MPM [59]. Due to the limited number of established patient-derived cell lines compared to large-scale targeted-sequencing studies of MPM patients, deviations in mutated cell lines and tumors were expected; however, they resemble different MPM genotypes useful for preclinical studies.

Further mutations in *DNMT3A* (10%) *and LRP1B* (10%) could be identified. These genes are known to drive stem cell expansion, hypomethylation, and proliferation in hematologic malignancies and lung cancer [60,61]. In MPM, *DNMT3A* alterations correlate with shorter survival [62]. Here, we detected a mutation in MUC19 in 60% of the patient-derived cell lines and in 30% of the originating tumors and a mutation in MUC4 in 40% of the patient-derived cell lines. These genes are involved in cell growth, disruption of tight junctions and adherens junctions, tumor progression, and blocking apoptosis [63]. There are currently multiple studies investigating mucins as a prognostic factor in cancer and the potential to target these molecules as a new treatment strategy [64,65]. However, the role of mucins in MPM is still not well understood.

To understand how common genomic alterations of *BAP1* and *CDKN2A* are conserved in patient-derived cell lines, we compared SNV and CNV frequencies across MPM tumors, patient-derived cell lines, and commercial cell lines used in this study and further commercial cell lines with genomic data listed in the cell model passport (Figure S8) [66]. All commercial cell lines have increased CNV and SNV frequencies of *BAP1* compared to patient-derived cell lines and tumors. The lack of *BAP1* was shown to increase genome instability and alter the response to microtubule targeting agents [67]. *CDKN2A* deletions are found in over 80% of commercial cell lines compared to below 20% of patient-derived cell lines and tumors. A total of 80% of MPM cell lines listed in this online repository are derived from pleural effusion. Pleural effusion cell lines harbor an increased frequency of *CDKN2A* alterations compared to data known from the clinics; hence, preclinical results involving this pathway should be considered with care [68]. *NF2* SNVs occur at similar frequencies in all cell lines and *TP53* SNVs and deletions can only be found in commercial cell lines from the cell model passport. From this model it will, however, remain unclear if the mutations in *BAP1* and *CDKN2A* were conserved from the originating tumor or acquired during passaging in the commercial cell lines. A large-scale study by Garman et al. identified *CDKN2A* alterations in 75% of melanoma cell lines compared to 24% in melanoma tumors [69]. Another study by Tam et al. published *CDKN2A* alterations in 75% of NSCLC cell lines compared to 38% in the originating tumors. The increased frequency of alterations in *BAP1* and *CDKN2A* gives commercial cell lines a growth advantage and may alter their overall drug response making patient-derived cell lines a better tool to study drug mechanisms and efficacy in MPM.

In the present study, patient-derived cell lines were more similar to the originating tumors according to their gene expression patterns in comparison to commercial cell lines. Several pathways are similarly regulated in tumor and patient-derived cell lines but either down- or upregulated in commercial cell lines. This includes the myogenesis, androgen response, coagulation, MYC targets, and EMT pathways. A comparison of patient-derived cell lines and commercial cell lines by Chernova et al. demonstrates a transcriptional upregulation of metabolic processes, cell signaling, and protein synthesis in commercial cell lines compared to a downregulation of apoptosis [16]. Here, we showed differential regulation of the same pathways, adding the transcriptome of the originating tumors, and identified higher similarities of patient-derived cell lines rather than commercial cell lines to the tumor material. These cell lines can be used for high-throughput drug screening approaches in a 2D in vitro system with high similarities to the originating tumors; more complex 3D in vitro systems and in vivo preclinical models can be further used for drug verification steps.

Previous studies revealed an association of EMT with cancer onset and invasiveness in MPM [70] leading to in vitro experiments and preclinical trials using EMT-targeting drugs and TGFβ inhibitors showed promising results in these studies [71]. However, a phase II study using galunisertib did not show clinical activity in patients with MPM [72]. Multiple genes involved in a more aggressive mesenchymal phenotype were upregulated in patient-derived cell lines, but with higher similarities compared to tumors than other commercial cell lines. This highlights that patient-derived cell lines would be of better use than cells derived from pleural effusion, as pleural effusion cell lines show a highly mesenchymal phenotype [73]. Human lung adenocarcinoma cells isolated from pleural effusion display increased chemo-resistance, potentially leading to false conclusions when used for drug assays compared to cells isolated from the primary tumors [74]. Furthermore, cancer cells isolated from pleural effusion of lung cancer patients include increased proportions of cancer stem cells and, thus, do not reflect the heterogeneity found in the primary tumors [75,76]. Our data demonstrate that the use of patient-derived cell lines with different genomic backgrounds, resembling the heterogeneity of MPM, would be preferable for preclinical studies to identify new treatment options for MPM targeting EMT.

In MPM, histone deacetylase inhibitors (HDACis) have shown promising results with the use of commercial cell lines [77,78] and in syngeneic MPM mouse models [79]. Based on these preclinical and limited phase 1 study results, a phase 3 study using the HDACi Vorinostat was conducted; however, no significant difference could be detected between the patients treated with this drug and the placebo group [80]. These conflicting results between preclinical and clinical studies could be explained by the use of highly passaged commercial cell lines in preclinical research. Our data on transcriptomics show an upregulation of HDACs in commercial cell lines compared to patient-derived cell lines. This increased expression of HDACs could lead to a false interpretation of in vitro data, with reduced effects of HDCAis in clinical trials.

Preclinical mouse models are widely used to translate in vitro results into a more complex living organism. However, pathogenesis mechanisms and the involved pathways differ between the human and mouse disease including processes like-cell duplication time, lifespan, and cancer susceptibility [81]. Here, we investigated transcriptomic differences of the syngeneic mouse mesothelioma model RN5 and the corresponding cell line to human tumors in order to better understand the additional value of using a mouse model for preclinical testing. *Bap1* and *Nf2* deficiencies have previously been described by Rehrauer et al., reflecting two of the most commonly altered genes in human MPM [42]. Gene sets involved in the P53 pathway, TGFβ signaling, and hypoxia were upregulated in RN5 tumors compared to the cell lines, indicating a similar regulation as in human patient-derived cell lines and tumors. However, EMT signaling is upregulated in RN5 tumors compared to human patient-derived cell lines and tumors. This upregulation of EMT genes in RN5 tumors has recently been shown by Wu et al., but no comparison to the in vitro cell line was included [82]. Recent studies showed that patient-derived xenograft mouse models might be a more appropriate tool to study EMT in lung adenocarcinoma and colorectal cancer [83,84]. The here-established patient-derived cell lines could further be of use for patient-derived xenograft (PDX) models, when implanted into immunodeficient mice to maintain the structure of the originating cancer tissue [85,86]. However, the major disadvantages of this model are the low tumor take (60%) and the lack of a tumor immune microenvironment. To overcome the issue of a missing immune system in xenograft models, humanized mice could be used; however, these models are still lacking for MPM [87].

Our results indicate a different regulation of multiple pathways of the murine RN5 model compared to human MPM tumors. Based on these data, this in vivo model can be of use; however, for pathway regulations or drug interactions specific considerations must be taken into account for each research question.

## 5. Conclusions

Here, we established and characterized low-passage-number patient-derived cell lines and demonstrated that these are a suitable tool for preclinical studies of MPM, as they closely resemble the genome and transcriptome of originating tumors. However, depending on specific pathways of interest, an appropriate model has to be chosen in regard to murine preclinical MPM models. These findings are of major relevance for the scientific community to use suitable models and avoid results which cannot be translated into clinical practice.

## Data Availability

The data for this study have been deposited in the European Nucleotide Archive (ENA) at EMBL-EBI under accession number PRJEB61319 (https://www.ebi.ac.uk/ena/browser/view/PRJEB61319, accessed on 19 April 2023).

## Supplementary Materials

The following supporting information can be downloaded at: https://www.mdpi.com/article/10.3390/cancers15102813/s1. Figure S1: Quality controls of patient-derived cell lines and isolated tumor cells for RNA sequencing; Figure S2: Gene expression comparison between missense mutated samples, samples containing gene deletions vs. unmutated samples; Figure S3: Differentially expressed genes in human MPM tumors compared to patient-derived cell lines; Figure S4: Gene set enrichment analysis (GSEA) for Hallmark gene sets for tumors vs. commercial cell lines; Figure S5: EMTome gene set analysis; Figure S6: Differentially expressed genes in murine RN5 tumors compared to the RN5 cell line; Figure S7: Venn diagram showing distribution of significantly differentially expressed genes across human and murine MPM samples; Figure S8: Mutation frequency of *BAP1*, *CDKN2A*, *NF2* and *TP53* in MPM tumors, patient-derived cell lines, and commercial cell lines used in this study and other commercial cell lines; Table S1: Characteristics of patients with tumors used of patient-derived cell line generation; Table S2: SNVs of all samples; Table S3: Top 100 up- and downregulated genes in tumors compared to patient-derived cell lines; Table S4: Top 100 up- and downregulated genes in tumors compared to established cell lines; Table S5: Top 100 up- and downregulated genes in patient-derived cell lines compared to established cell lines; and Table S6: Top 100 up- and downregulated genes in RN5 cell line compared to tumors; Table S7 Western blot membranes and quantifications.
