# Supplementary material for: Genomic and Transcriptomic Analyses of Malignant Pleural Mesothelioma (MPM) Samples Reveal Crucial Insights for Preclinical Testing
Source: Cancers (Basel). 2023 May 18;15(10):2813. doi: 10.3390/cancers15102813 (PMC10216513; doi:10.3390/cancers15102813)
Supplement: Supplementary file 1 [file cancers-15-02813-s001.zip › Manuscript Laure_Supplementary Figures.pdf]

cancers

Article

Genomic and Transcriptomic Analyses of Malignant Pleural Mesothelioma (MPM) Samples Reveal Crucial Insights for Preclinical Testing

Alexander Laure [1,2], Angelica Rigutto [1,2], Michaela B. Kirschner [3], Lennart Opitz [4], Linda Grob [5,6], Isabelle Opitz [2,3], Emanuela Felley-Bosco [2,7], Stefanie Hiltbrunner [1,8,†] and Alessandra Curioni-Fontecedro [1,8,9,*,†]

[1] Faculty of Science and Medicine, University of Fribourg, CH-1700 Fribourg, Switzerland
[2] Faculty of Science, University of Zurich, CH-8006 Zurich, Switzerland
[3] Department of Thoracic Surgery, University Hospital Zurich, CH-8091 Zurich, Switzerland
[4] Functional Genomics Center Zurich, Swiss Federal Institute of Technology, University of Zurich, CH-8057 Zurich, Switzerland
[5] NEXUS Personalized Health Technologies, ETH Zurich, CH-8092 Zurich, Switzerland
[6] Swiss Institute of Bioinformatics, CH-1015 Lausanne, Switzerland
[7] Laboratory of Molecular Oncology, Department of Thoracic Surgery, University Hospital Zurich, CH-8091 Zurich, Switzerland
[8] Department of Medical Oncology and Haematology, University Hospital Zurich, CH-8091 Zurich, Switzerland
[9] Department of Oncology, HFR Fribourg-Hôpital Cantonal, CH-1708 Fribourg, Switzerland
* Correspondence: alessandra.curioni-fontecedro@h-fr.ch; Tel.: +41-26-306-22-60
† These authors contributed equally to this work.

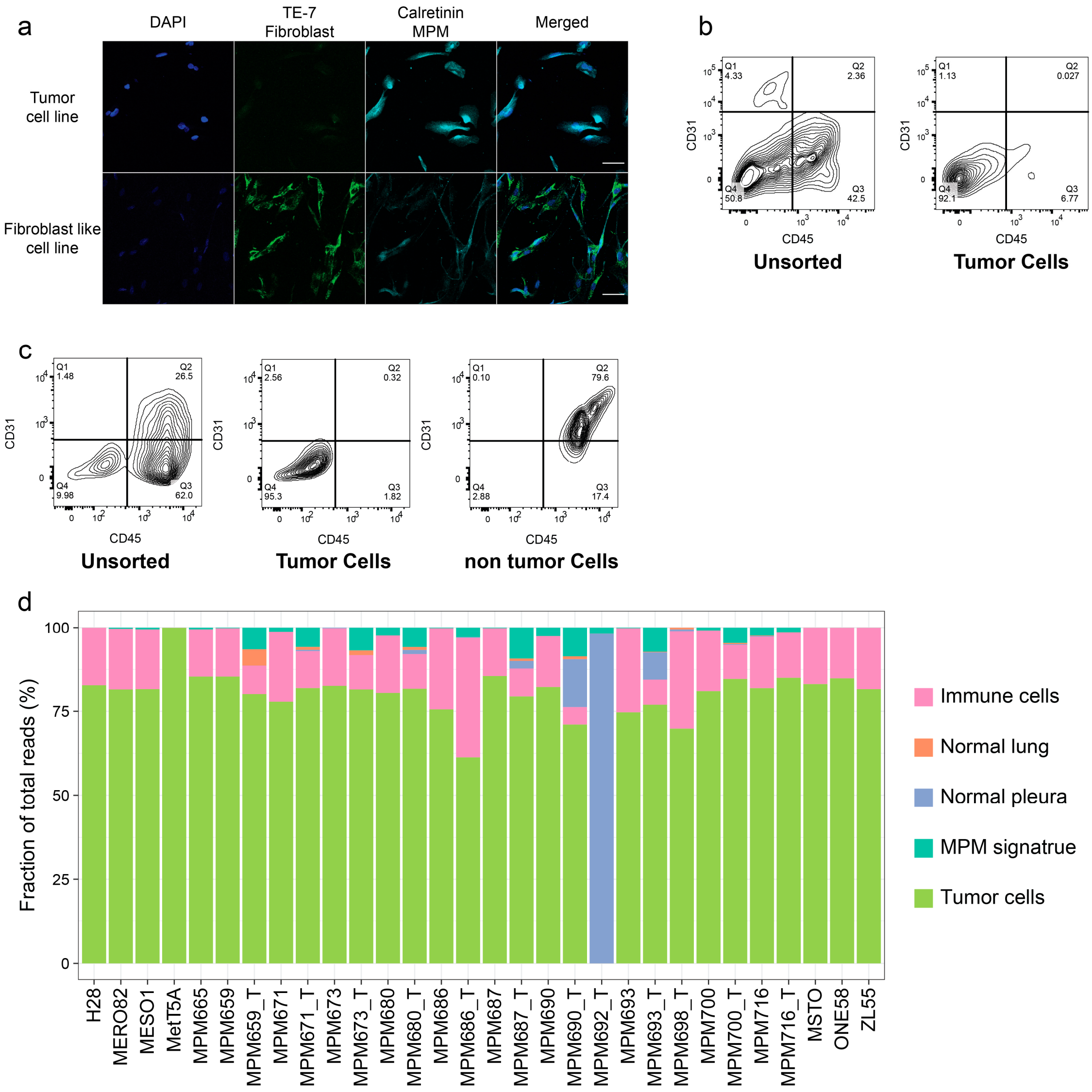

Figure S1. Quality controls of patient-derived cell lines and isolated tumor cells for RNA sequencing. (a) ICC staining of patient-derived cell lines for DAPI (blue), TE-7 (green), a lung specific fibroblast marker and Calretinin (cyan), a MPM marker, showed no fibroblast contaminations in 10 individual MPM cell lines. Representative figure of MPM655. (50µm scale bar) Purity of the tumor cell fraction from (b) human and (c) mouse tumors after depletion of CD45+ immune cells and CD31+ endothelial cells using the Miltenyi tumor cell isolation kit, (d) Deconvolution of bulk sequencing data using Granulator. The sequencing data was deconvoluted using reference gene expression data for immune cell subtypes, healthy lung and pleura. For the final deconvolution, the Non-negative least squares (nnls) algorithm was used.

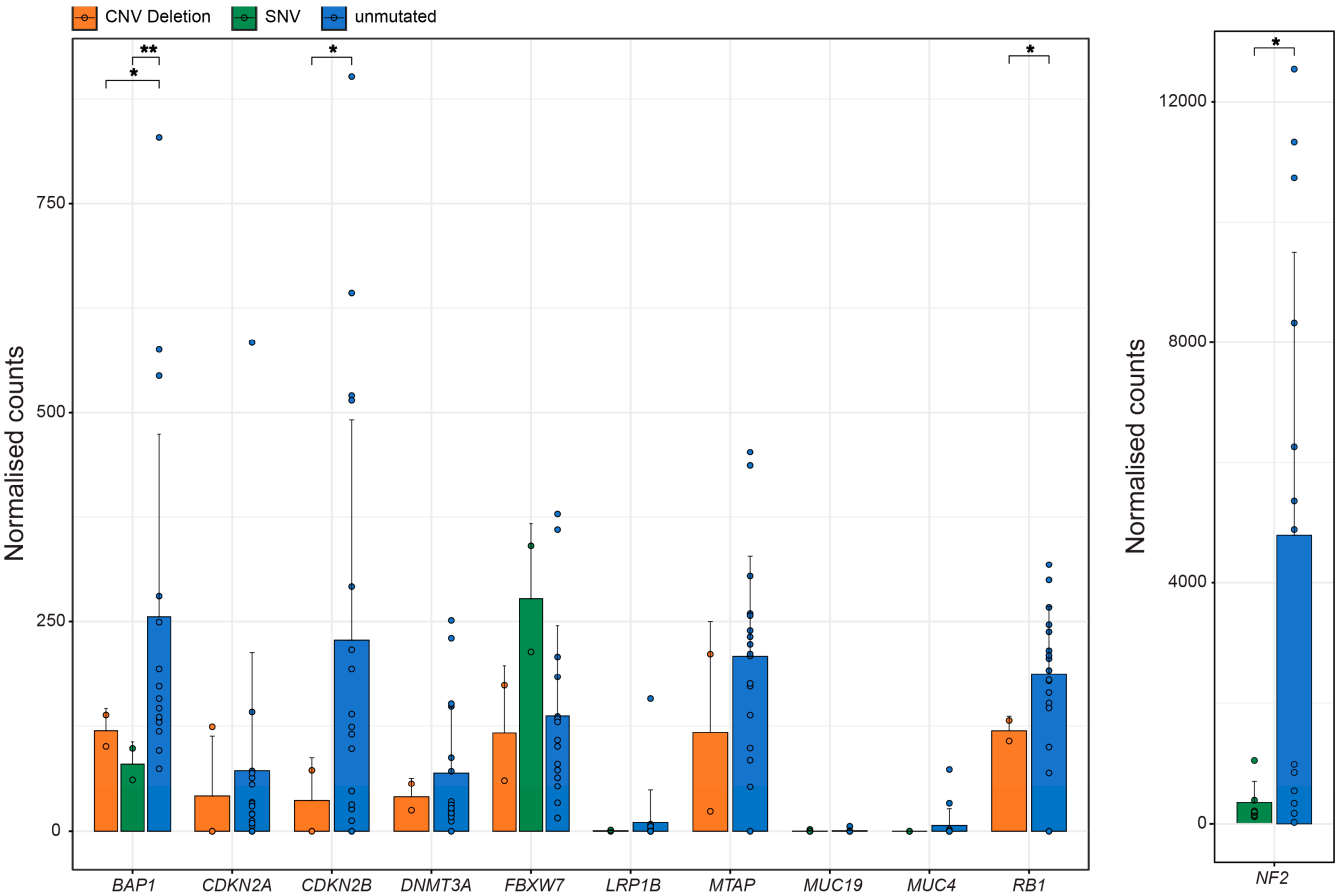

Figure S2. Gene expression comparison between missense mutated samples, samples containing gene deletions vs. unmutated samples. mRNA is shown as normalized counts of whole mRNA sequencing. Significance was calculated using a t.test and p-values were adjusted using Bonferroni corrections. ****<0.0001, ***<0.001, **<0.01, *<0.05.

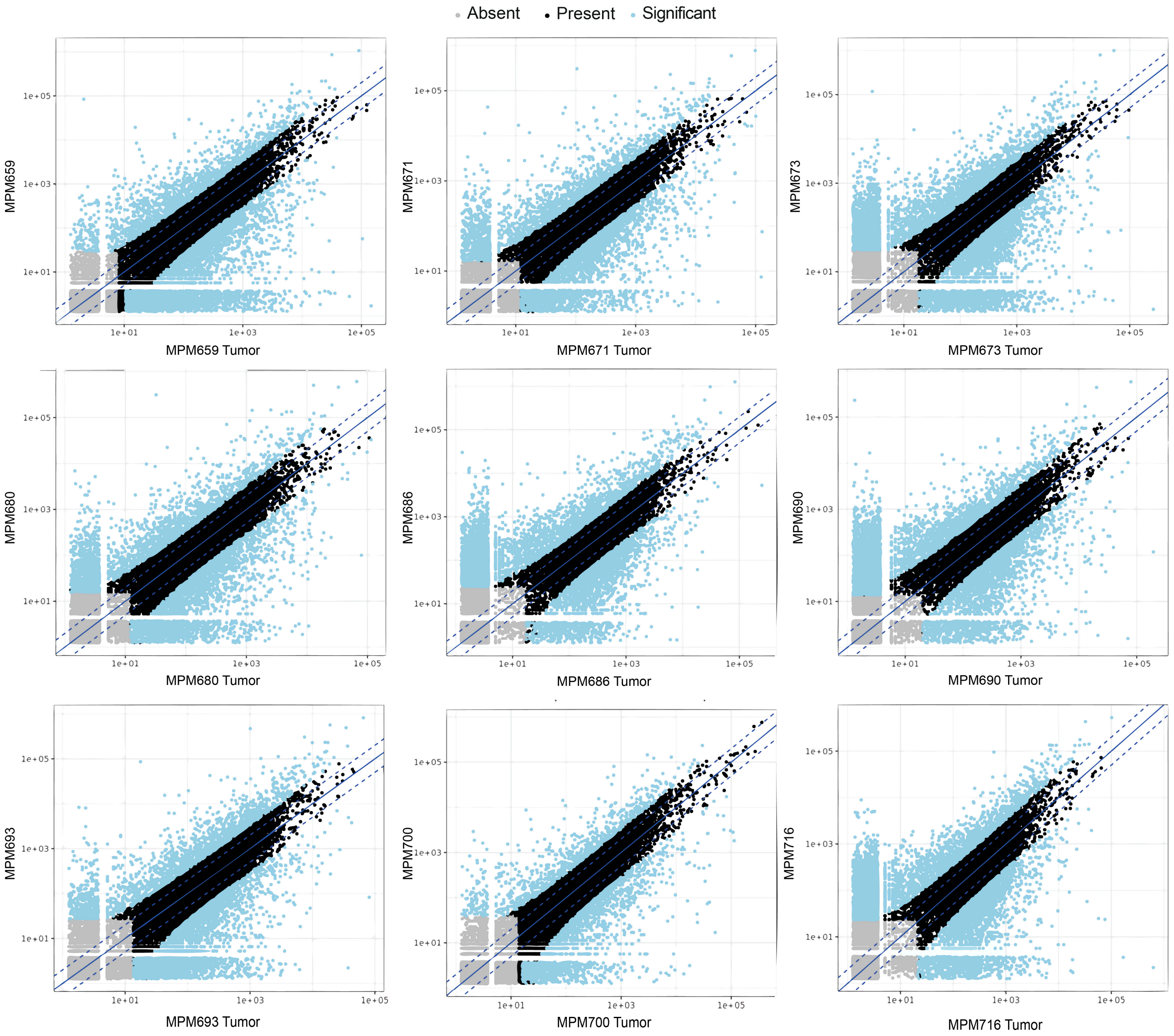

Figure S3. Differentially expressed genes in human MPM tumors compared to. patient-derived cell lines. Volcano plot of differentially expressed genes of matching patient-derived cell lines and corresponding fresh tumors. p<=0.01 and log2 ratio <=0.5

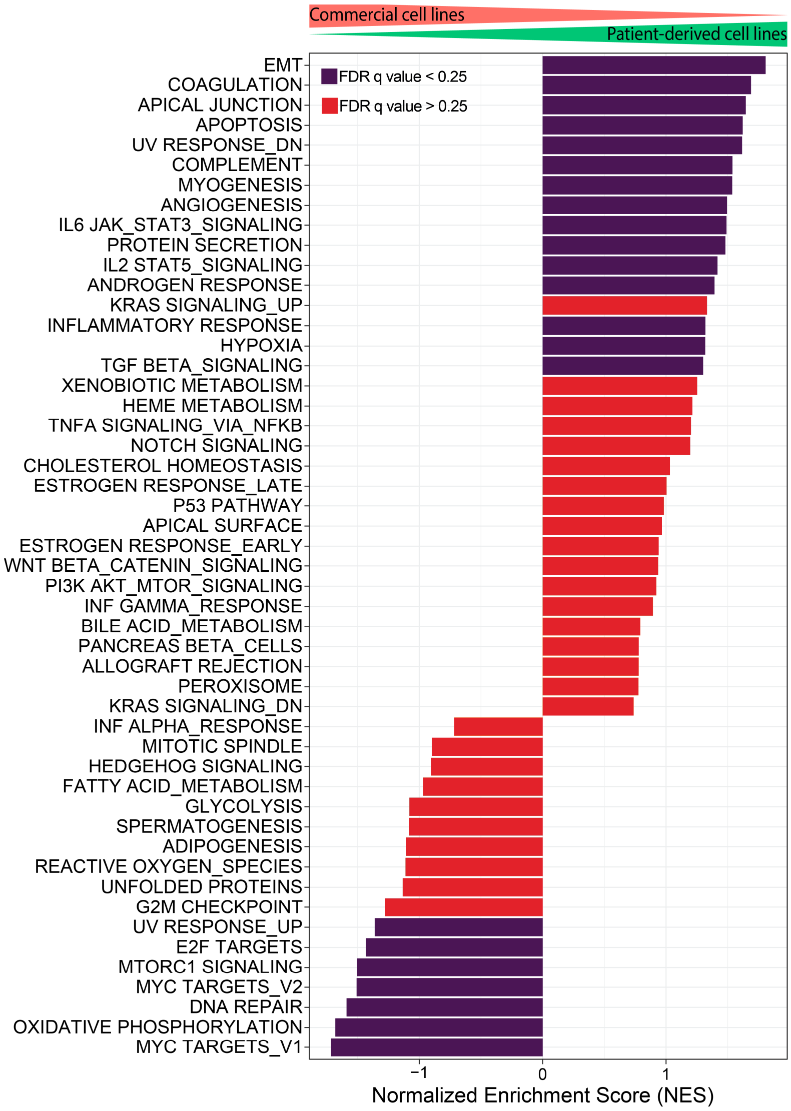

Figure S4. Gene set enrichment analysis (GSEA) for Hallmark gene sets for commercial vs. patient-derived cell lines. Blue bars indicate significant enrichment between groups with FDR q values < 0.25. Red bars indicate non-significant FDR q values > 0.25. A positive Normalized Enrichment Score (NES) indicates enrichment in patient-derived cell lines, a negative NES indicates enrichment in commercial cell lines.

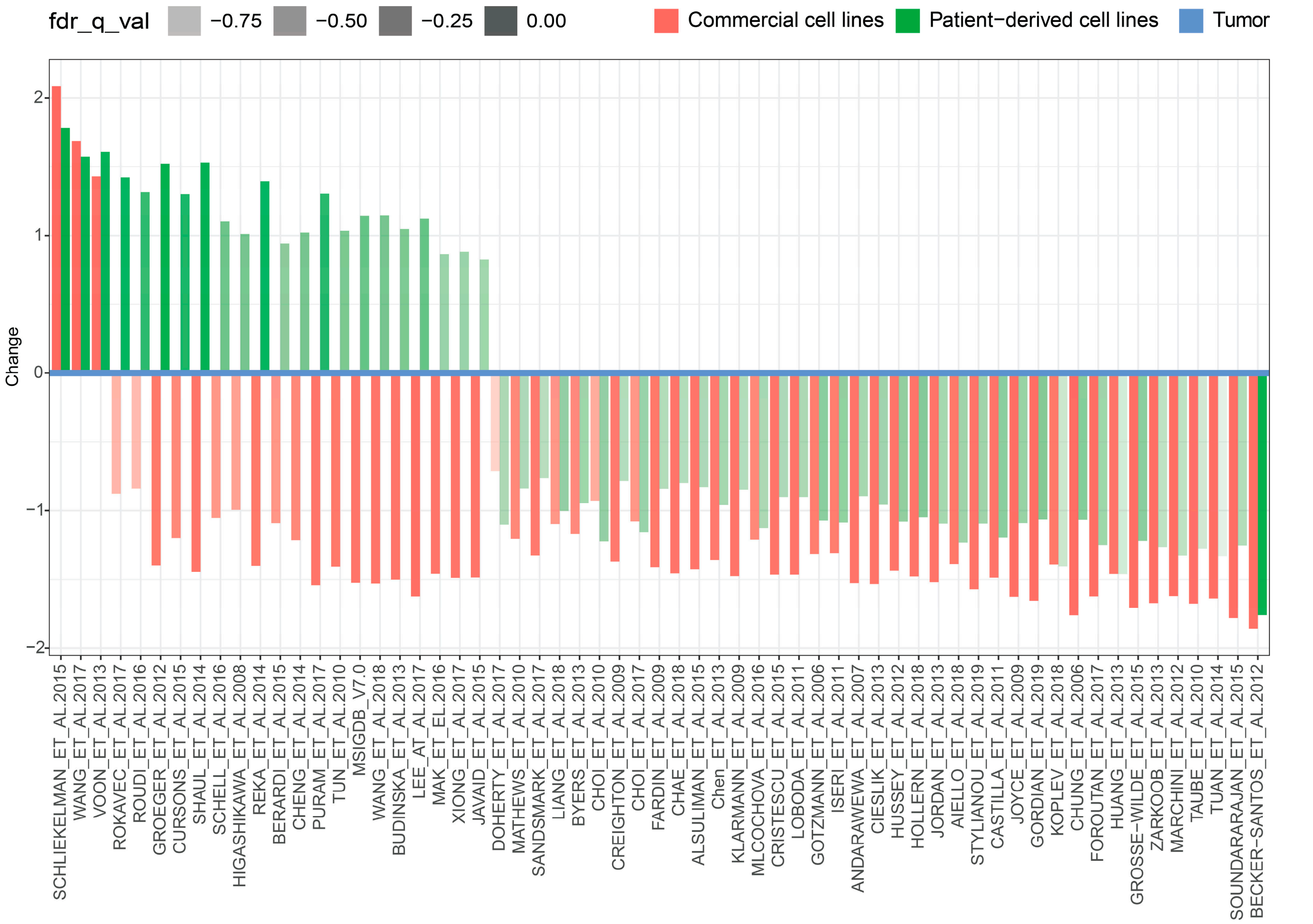

Figure S5. EMTome gene set analysis. EMT gene sets from 81 publications were retrieved from the EMTome. Color-intensities represent the significance (FDR q value) and NES scores were normalized to the expression in primary tumors. Positive values show an increase compared to tumors and negative values a decrease compared to tumors.

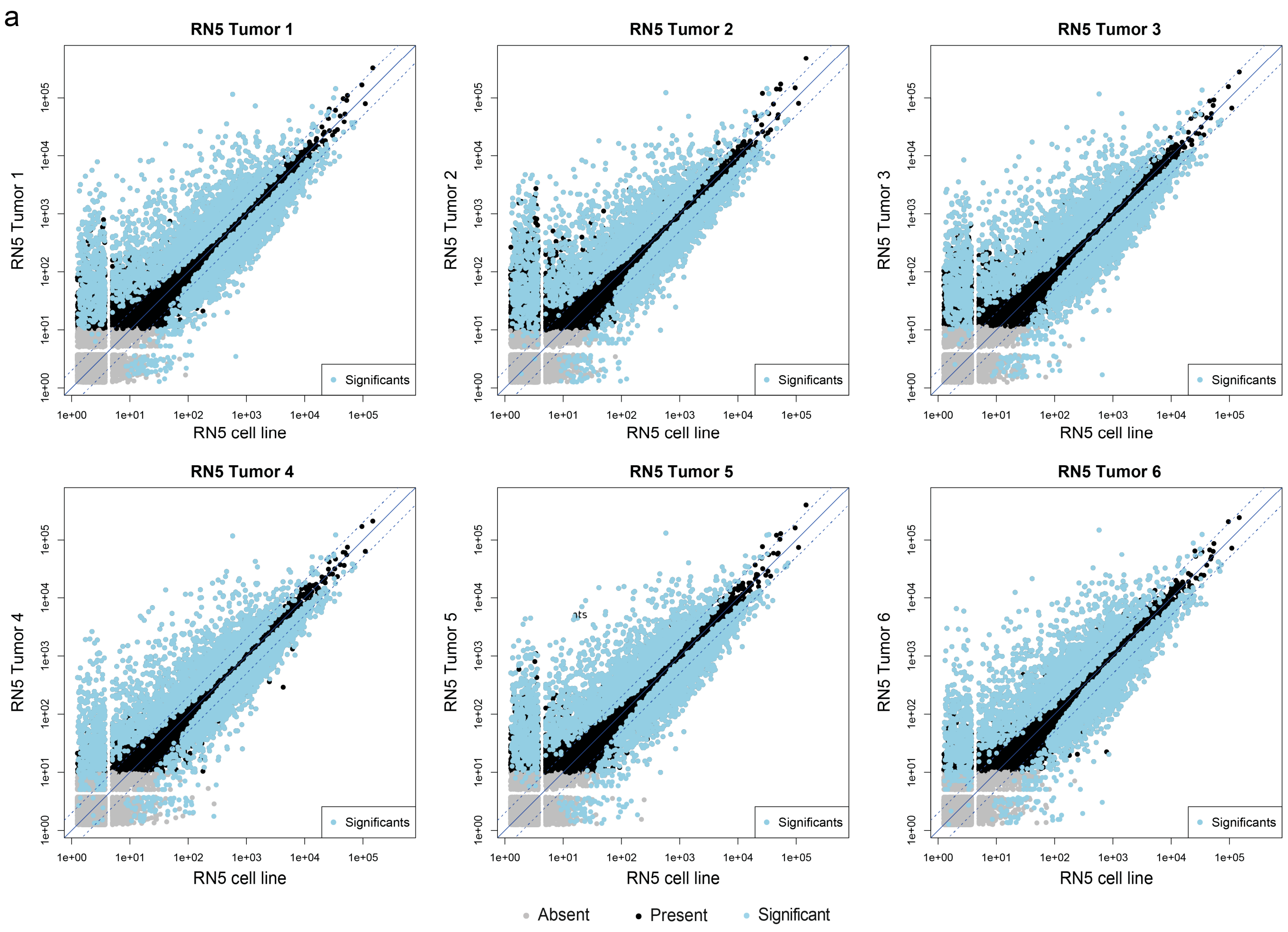

Figure S6. Differentially expressed genes in murine RN5 tumors compared to the. RN5 cell line. Volcano plot of differentially expressed genes of the RN5 cell line and. RN5 tumors. p<=0.01 and log2 ratio <=0.5

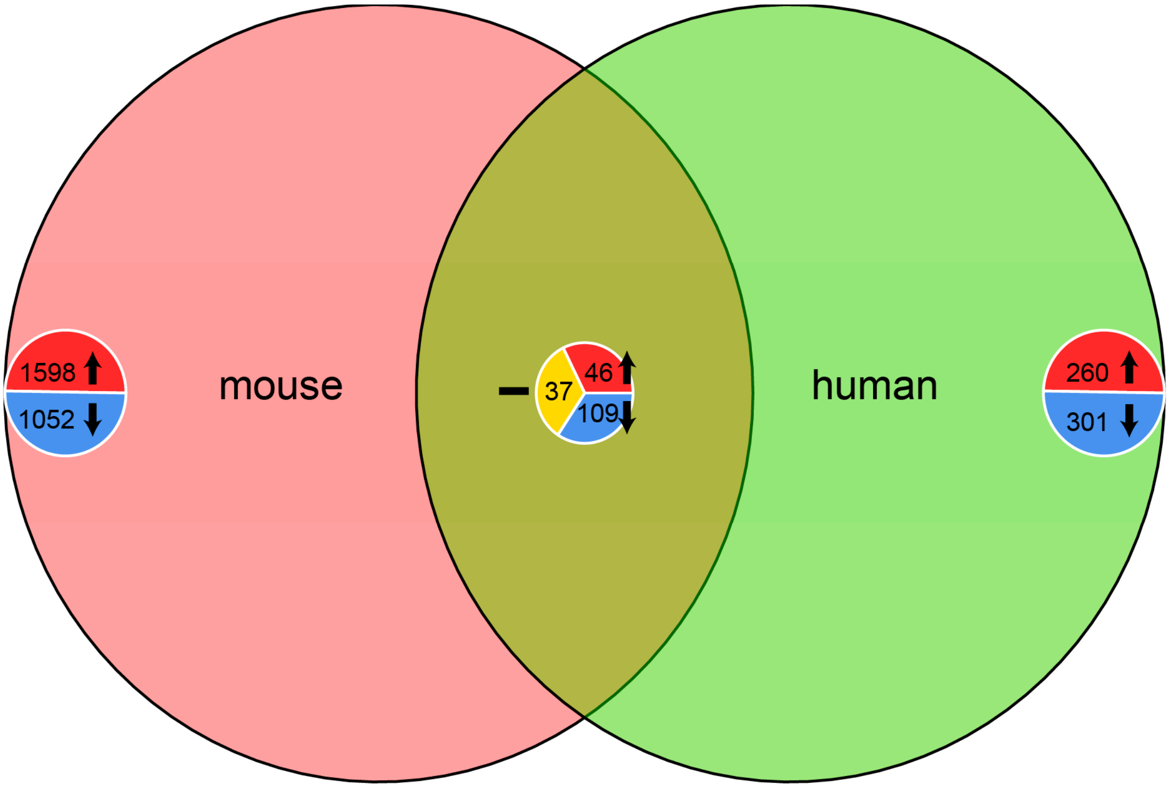

Figure S7. Venn diagram showing distribution of significantly differentially expressed genes across human and murine MPM samples. Up- (red), and downregulated (blue) genes in tumors and. cell lines were analyzed for each species to identify identically regulated genes. Genes in the corresponding circles were only differentially expressed in this species (left and right) and genes identified in both species (middle) included genes that are differentially regulated in each species (yellow). Cutoffs of p <0.00001 and log2_expression_ratio between tumor and cell line of 2 were used.

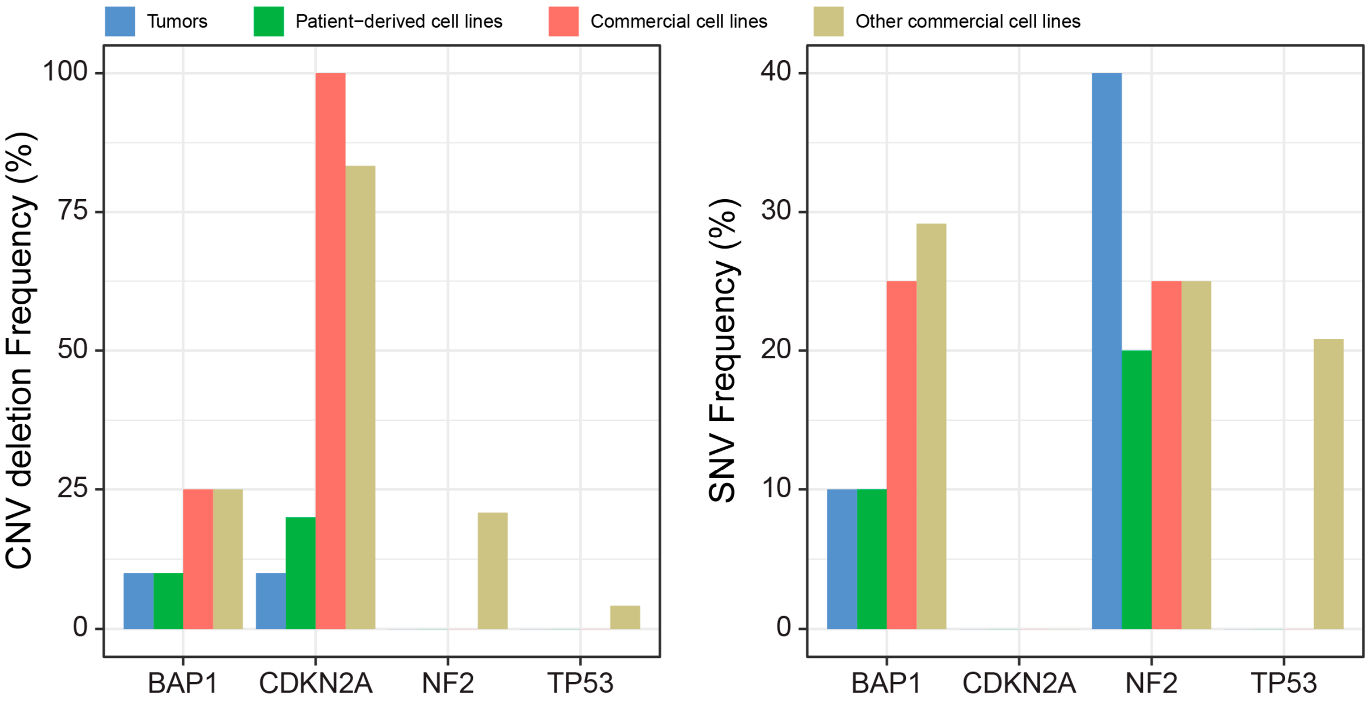

Figure S8. Mutation frequency of BAP1, CDKN2A, NF2 and TP53 in MPM tumors, patient-derived cell lines, commercial cell lines used in this study and other commercial cell lines. CNV and SNV mutation frequencies in % for MPM tumors (blue), patient-derived cell lines (green), commercial cell lines used in this study (red) and other commercial cell lines with genomic data listen in the cell model passport (yellow).
